# Haplotype GWAS in Swedish warmblood horses for conformation and jumping traits

**DOI:** 10.1093/jas/skag109

**Published:** 2026-04-10

**Authors:** Michela Ablondi, Susanne Eriksson, Åsa Gelinder Viklund, Sofia Mikko

**Affiliations:** Department of Veterinary Science, Università degli Studi di Parma, 43126 Parma, Italy; Department of Animal Biosciences, Swedish University of Agricultural Sciences, 75007 Uppsala, Sweden; Department of Animal Biosciences, Swedish University of Agricultural Sciences, 75007 Uppsala, Sweden; Department of Animal Biosciences, Swedish University of Agricultural Sciences, 75007 Uppsala, Sweden; Department of Animal Biosciences, Swedish University of Agricultural Sciences, 75007 Uppsala, Sweden

**Keywords:** association study, equine, genomics, haplotype, performance

## Abstract

The aim of this study was to better understand the genomic architecture behind performance-related traits in sport horses. In this study, we conducted a haplotype-based genome-wide association study (GWAS) for 36 conformation and free jumping phenotypes recorded during routinely conducted young horse evaluation tests involving 380 Swedish Warmblood (SWB) horses. The horses were evaluated by expert judges using both traditional and linear evaluation systems. All samples were genotyped using the 670K Affymetrix^®^ Axiom^®^ Equine Genotyping Array, haplotypes were first phased, and haplotype blocks were calculated for a total of 78,000 haplotypes. To assess the association between the haplotypes and studied traits, a single-trait linear mixed model was used, correcting for sex and the date-location in which the evaluation took place. In the analysis, a total of 11 haplotype blocks were found to be significantly associated with a total of six traits: height at withers, the conformation traits hooves and correctness in movement, and the free jumping traits technique: haunches, carefulness, and distance estimation. In the proximity of those haplotypes (windows size ± 500 kb), 33 protein-coding genes, 31 IncRNAs, and one miRNA were found. Within those regions, key candidate genes were located such as *LCORL* and *KHDRBS3*, associated with body size and growth, as well as *COL12A1*, *MYO6*, and *FILIP1*, involved in musculoskeletal development and muscle elasticity and strength. The haplotype-based GWAS approach proved to be a useful method since it helped in the detection of aggregated genetic effects. Future studies with larger sample sizes and using novel tools for objective phenotyping will be essential to further investigate the genetic mechanisms behind sports performance.

## Lay Summary

The Swedish Warmblood is a sport horse breed, valued for its performance, rideability, and temperament. To improve breeding, it is important to understand the genetic background of traits linked to sport performance. However, these traits are influenced by many genes, making them difficult to study. In this project, we analyzed the DNA of 380 young Swedish Warmblood horses, all evaluated by expert judges for conformation and free jumping ability. Using a haplotype-based genome-wide association study (GWAS), we grouped genetic markers into blocks (haplotypes) and tested their relationship with 36 traits. We found 11 genetic regions linked to 6 conformation traits: height at the withers, hoof quality, correctness of movement, and three free jumping traits (technique in the haunches, carefulness, and distance estimation). Within these regions, we identified 33 coding genes and several non-coding RNA genes that may play a role in defining horse performance. This haplotype-based approach gave novel insights into the genetic background of sport performance-related traits in horses. Future studies with more horses and advanced digital tools for measuring traits will help to further reveal the complex genetic basis of sport performance in horses.

## Teaser

This study identifies chromosome regions linked to key conformation and free jumping traits in Swedish Warmblood horses through a haplotype GWAS approach, providing new insights into the complex genomic basis of sport performance.

## Introduction

In the last decades, the sport horse breeding sector has been challenged with the task of selecting horses for highly ambitious sport competitions. The Swedish Warmblood Association (SWB) fits into this scenario with the breeding goal to produce internationally competitive warmblood horses in terms of rideability and performance-oriented temperament and with excellent gaits and/or jumping ability (Ablondi et al. 2019a; Ablondi et al. 2019b). These challenges have been tackled by the breeding association via intensive phenotypic recording systems as a basis for estimating breeding values for sport traits (Viklund et al. 2008; Viklund et al. 2010; Viklund and Eriksson 2018). However, those initiatives still lack a full understanding of the genomic background of the traits under selection. By knowing the genomic background of sport-related traits, the selection process might benefit from the introduction of marker-assisted selection (MAS) or implementation of genomic selection (GS). Genome-wide association study (GWAS) has long been used as a tool to identify genomic regions associated with phenotype variations to further understand the genetic background of the traits (Begum et al. 2012). Over the last decade, GWAS have been performed in horses, especially in the context of harmful diseases such as osteochondrosis (Corbin et al. 2012), laryngeal neuropathy (Dupuis et al. 2011), and insect bite hypersensitivity (Schurink et al. 2012; Shrestha et al. 2020), although without finding any causative variants. However, its application in sport performance in warmblood riding horses is still limited with few recent studies performing either single-step GWAS (ssGWAS) or SNP-by-SNP GWAS for sport performance-related traits (Brard and Ricard 2015; Ricard et al. 2020; Nazari‐Ghadikolaei et al. 2025). There are some known genetic variants associated with performance in horses, such as the polymorphism in the *MSTN* gene, which has been proposed as a predictor of speed in Thoroughbreds (Hill et al. 2010; Hill et al. 2010; Hill et al. 2012). Specifically, Thoroughbred horses homozygous for the derived allele are developing quicker at an earlier age, have more muscles, and do better in short-distance races. The *MSTN* variant was also associated with better suitability as a sprinter in the Quarter Horse and linked with a higher proportion of fast-twitch muscle fibers in this breed (Petersen et al. 2013, 2014). Another example of successful identification of genes associated with performance is the *DMRT3* gene, which has been found to affect the pattern of locomotion in horses (Andersson et al. 2012). Polymorphisms in the *DMRT3* have been associated with the ability to perform alternate gaits and to harness racing performance (Jäderkvist Fegraeus et al. 2017; Staiger et al. 2017). A recent study found a single quantitative trait kloci (QTL) for conformation, which also influences lateral gait quality in Icelandic horses (Rosengren et al. 2021). A GWAS in Yili horses identified 127 candidate genes associated with racing performance traits, including 50 significant ones (Wang et al. 2024). Notably, genes such as *CNTN6*, which is involved in motor coordination, *NIPA1*, which is involved in neuronal development, and *DCC* which has a role in dopamine pathway maturation, were linked to speed traits. Moreover, genes with a link to neurological pathways like *SHANK2* and *KCNIP4* were associated with ranking score traits. In endurance horses, transcriptomic analyses have revealed differentially expressed genes (DEGs) related to endurance runs (Reißmann et al. 2023). For instance, after long-distance runs, genes such as *ACOD1*, *CCL5*, *CD40LG*, *FOS*, *IL1R2*, *IL20RA*, and *IL22RA2* were upregulated, indicating stress response and metabolic processes owing to intensive performance. These genes may therefore serve as candidates for monitoring and evaluating performance in endurance horses. Furthermore, recent research has identified a frameshift mutation in the *STAU2* gene and regulatory elements in the *RELN* gene that influence gait performance in Icelandic horses (Sigurðardóttir et al. 2023). These genetic variants were associated with the ability to perform specific gaits like tölt (a four-beat lateral ambling gait typical of Icelandic horses) and pace, as well as with traits related to trainability and precocity.

Nevertheless, the genomic background of performance traits in warmblood sport horses is still mainly uncovered. Sport performance traits are polygenic traits, as they are typically governed by small contributions of numerous variants across the genome, possibly together with some QTLs with larger effects. In addition, complex traits such as sport performance outcome are caused not only by the underlying genetic variation but also due to several environmental effects (reviewed by Raudsepp et al. 2019). Recently, it has been shown that the employment of haplotypes instead of SNPs may assist a GWAS to establish marker-phenotype associations (Lorenz et al. 2010; Barendse 2011; Bovo et al. 2021). Although haplotype-based GWAS does not inherently overcome the limitations of small sample size or polygenic trait architecture, it has been shown that it might help in the detection of aggregated genetic effects, particularly for QTL with low minor allele frequency, and explain a larger proportion of phenotypic variance than single-SNP approaches (Lorenz et al. 2010; Barendse 2011; Bovo et al. 2021). Thus, in this study we utilized a haplotype-based GWAS approach of 36 phenotypes in 380 SWB horses with the aim to further dissect the genomic architecture behind show jumping and conformation traits.

## Materials and methods

This study did not involve any invasive procedures or handling of horses. Hair samples from SWB horses were originally collected for parentage analyses by the SWB Association and stored in the biobank at the Animal Genetics Laboratory, Swedish University of Agricultural Sciences (SLU). The use of these pre-existing samples for research was approved by the Swedish Warmblood Association, and owners’ consent was given in connection with registration of the horse. As no additional material was collected, ethical approval was not applicable.

### Phenotypes

A total of 380 SWB horses (182 males and 198 females) born in 2010–2011 were included in this study, and they were assessed during routinely conducted young horse tests at the age of three years (Viklund and Eriksson 2018). The assessments included three main categories of traits: conformation, free-jumping, and gaits as previously analyzed in other studies (Ablondi et al. 2022; Bonow et al. 2024; Nazari‐Ghadikolaei et al. 2025). For the scope of this study, we focused on conformation and free jumping traits. The horses were judged by teams of two people, one expert in jumping and one expert in conformation and movements, evaluating the animals using both a traditional 10-point evaluating system according to the breeding goal as well as a linear descriptive 9-point scale between biological extremes. For linear scoring, a few horses did not have the data available for certain traits, thus, the exact number of horses per trait varied from 351 to 272. In addition, the height at withers in cm was also measured during the young horse test. In this study, a total of 36 scored phenotypes were analyzed: one measure (height at withers), five traditional evaluating traits, one total score, which is the sum of traditional evaluating traits of relevance for show jumping, and 29 linearly scored traits. In brief, the 380 SWB horses included in this study were selected to represent both show jumping and non-show jumping horses with high and low performance scores as described in a previous study (Ablondi et al. 2019b).

### Genotyping, quality control, and haplotype phasing

For the 380 SWB horses, DNA was extracted from hair roots as further described in (Ablondi et al. 2019a; Ablondi et al. 2019b). All samples were genotyped using the 670K Affymetrix^®^ Axiom^®^ Equine Genotyping Array (Thermo Fisher Scientific, Santa Clara, CA, USA) at SciLifeLab (Uppsala, Sweden) and remapped from the former EquCab2 to EquCab3 (Kalbfleisch et al. 2018) reference genome using a Python script, as described in (Beeson et al. 2019). Only SNPs located on the 31 autosomes were retrieved and used in this study (606,287 SNPs). The quality control (QC) was performed in PLINK (v1.9) (Purcell et al. 2007) by removing SNPs and horses with a call rate lower than 0.90, SNPs with minor allele frequency below 0.05, and Hardy–Weinberg equilibrium (HWE) deviation with *P* < 0.000001. Genotypes were then phased using SHAPEIT v.2 (Delaneau et al. 2008). Although we did not check phasing accuracy based on family data, previous studies have shown that SHAPEIT achieves low switch error rates when inferring haplotypes from genotype arrays (Delaneau et al. 2011). Haplotype blocks were defined using the R package GHap 1.2.2 (Utsunomiya et al. 2016). Haplotype blocks were then constructed using ghap.blockgen with a fixed-SNP sliding-window approach. Specifically, the final GWAS analyses were based on non-overlapping windows of 5 consecutive SNPs (window size = 5, slide = 5, unit = “marker”). We evaluated alternative block definitions (eg 10 SNP/kb windows). However, larger blocks increased haplotype fragmentation and reduced haplotype frequencies, leading to more sparse classes; therefore, we retained 5-SNP blocks as the best compromise between capturing local LD and maintaining sufficient haplotype counts for association testing. The haplotype blocks were exported as *tped* file format, where haplotype allele counts 0, 1, and 2 were recoded as NN, NH, and HH genotypes (H = haplotype allele and N = NULL meaning all other alleles), as if haplotypes were bi-allelic markers. A regular ped and map format was obtained with PLINK (v1.9) filtering out haplotypes presenting a MAF < 0.01 (Bovo et al. 2021).

### Haplotype genome wide association studies

GWAS were performed for all the 36 traits in GenABEL R package (R Development Core Team 2011). A polygenic model approach was used to account for population stratification (Gmel et al. 2019), as genetic divergence due to sport discipline was found in previous work (Ablondi et al. 2019a). Specifically, a genomic relationship matrix (GRM) was constructed using autosomal markers based on identity-by-state (IBS) sharing, weighted by allele frequencies. Association testing was performed using the mmscore() function, which performs fast GWAS using a two-step approach: it first fits a mixed linear model to estimate variance components while correcting for population stratification and relatedness using the genomic kinship matrix and then tests each marker (haplotype allele count) individually using a score test. All traits were assumed to follow a Gaussian distribution. No pedigree-based relationship matrix was used. Lambda (λ) values were checked to be between 0.95 and 1.05. In the statistical model the following fixed effects were included: sex (female or male) and test-event, which is the combination of the date and the location where the evaluation took place. Haplotypes passing Bonferroni correction (0.05/number of haplotype blocks [*P* < 6.4 × 10 − 7]) were considered as significantly associated ones. To further evaluate any signs of potential false positive-associated signals, we examined the quantile-quantile (Q-Q) plots for the inflation of small *P*-values, and we calculated the λ value. The estimated regression coefficient represents the effect per additional haplotype copy under an additive model and should not be interpreted as a classical SNP allele substitution effect. The ggstatsplot package in R was used to evaluate statistically significant differences via Welch ANOVA and visualize the mean differences between the most significant haplotype compared to the others (Patil 2021).

### Functional annotation

The Ensembl gene annotation EquCab3 (Aken et al. 2016) was used to identify genes residing within regions extending 500 kb up and downstream each significant haplotype. This was done to include potential effects of regulatory changes on loci at some distance and to reduce the risk of excluding the outermost parts of the associated haplotypes. We also checked if any of the significantly associated regions were in the vicinity of annotated QTLs based on the HorseQTL database (Hu et al. 2022).

## Results

### Quality control and haplotype blocks

All 380 SWB horses passed the QC, and 389,997 SNPs were phased and used to create haplotype blocks. A total of 78,000 haplotype blocks were found with an average of 5.6 haplotype combinations per block.

### Traditional evaluating traits and related haplotypes

**Figure 1:**
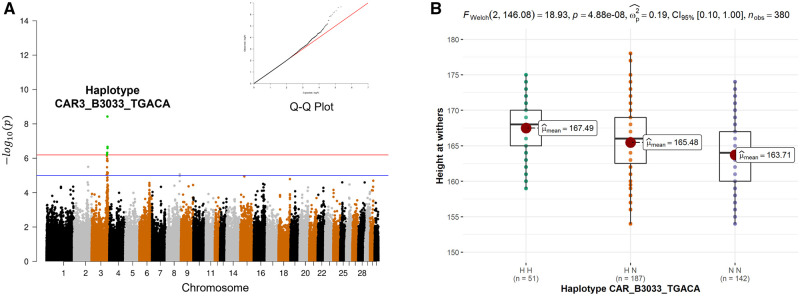
Genome-wide association study with haplotype blocks for the height at withers. (A) Manhattan plot with bottom line representing the suggestive significance threshold (*P* < 10^−6^) and top line the significance threshold after Bonferroni correction (*P* < 6.4 × 10^−7^). The plot on the right-hand corner shows the quantile–quantile (Q–Q) plot with the observed plotted against the expected *P*-value. (B) Height at withers in cm for horses with the most significant haplotype (H) on chromosome 3, compared with other haplotypes (N) treated as bi-allelic variants. The red dot indicates the average value. Above the plot the results from the F-welch statistics are reported.

Descriptive statistics of the traditional evaluating traits is presented in Table 1, where it is shown that for the free jumping traits the whole scale was used by the judges, in contrast to the conformation traits, where the range was from score 5 to 10. The average height at withers was 165.1 cm (SD ±4.34 cm). Five haplotypes on ECA3 were significantly associated (*P* < 6.17E-07) with height at withers (Table 2 and Figure 1), the remaining traditional evaluating traits, no haplotypes were found to be significantly associated. The haplotype (CAR3_B3033_TGACA) was strongly associated with height at withers and had a beta regression coefficient equal to 1.81 (± 0.31), and the CAR3_B2992_TTCTG included the *LCORL* gene. Horses’ homozygote for this haplotype was on average 3.78 centimeters taller than horses not carrying this haplotype, as shown in (Figure 1 [*P*-value <0.01]). A total of 33 genomic elements (including protein coding genes, pseudogenes, and ncRNA) resided within ± 500 kb of the five significantly associated haplotype regions with height at withers: 18 protein-coding genes, two pseudogenes, 11 IncRNA, one miRNA, and one snRNA (Supplementary Table S1).

**Table 1:** Assessment scale, mean and standard deviation (SD) of the phenotypes analyzed for 380 horses using the traditional evaluating system and divided by trait type.

Traditional evaluating traits					
Trait type	Variable	Mean	SD	Min	Max
**Conformation**	Correctness of legs	7.20	0.63	5	9
	Type	7.80	0.70	6	10
	Head, neck and body	7.70	0.61	6	9
**Free jumping**	Jumping technique	6.90	1.78	1	10
	Jumping temperament	6.90	1.89	1	10
**Total score**	Total Score Show Jumping	44.0	4.66	31	54
**Measure**	Height at withers (cm)	165.1	4.34	154.0	178.0

**Table 2:** Five haplotype on ECA3 significantly associated with height at withers in the present study. **N_HH_** denotes the number of individuals carrying the HH haplotype. Annotated protein coding genes found from the haplotype ± 500 kb are also shown.

Haplotype	ECA	Centered position	*P*-value	Beta regression coefficient (SE)	N_HH_	Annotated protein coding genes
**CAR3_B3033_TGACA**	3	108524170	4.98E-09	1.81 (0.31)	51	*LDB2, TAPT1*
**CAR3_B3060_TAGTC**	3	109359454	2.52E-07	−2.06 (0.40)	18	*TAPT1, PROM1, FGFBP1, FBXL15, CD38, BST1, FAM200B, CC2D2A, C1QTNF7, ENSECAG00000034729, ENSECAG00000051073*
**CAR3_B3051_CGTTT**	3	109081691	3.02E-07	1.98 (0.39)	15	*TAPT1, PROM1, FGFBP1, CD38, BST1, FAM200B, FBXL5, CC2D2A, C1QTNF7*
**CAR3_B2992_TTCTG**	3	106983820	4.78E-07	1.87 (0.37)	21	*LCORL, ENSECAG00000057490*
**CAR3_B2952_TGCCA**	3	105634956	6.17E-07	−2.31 (0.46)	8	*KCNIP4, PACRGL, SLIT2, MIR218, ENSECAG00000057886, ENSECAG00000003040*

### Linear descriptive traits and related haplotypes

Descriptive statistics of the linearly described traits are presented in Tables 3 and 4. Similarly, to what was shown for traditional evaluation, the judges tended to not fully use the whole scale, and the range was for most conformation traits between scores 3 and 7 (Table 3). In contrast, for several jumping traits a wider range was used (Table 4). A total of six haplotypes were found to be significantly associated with five linearly described traits: two conformation traits (hooves and correctness in movement) and three free jumping traits (technique: haunches, carefulness, and distance estimation). All 38 genomic elements, including IncRNA, miRNA, snRNA, and pseudogenes, are presented in Supplementary Table S2.

**Table 3:** Assessment scale, number of observations, mean and standard deviation (SD) for the linear descriptive trait for conformation.

Variable	Scale	Horses	Mean	SD	Min	Max
**Type**	Refined—Heavy	372	4.70	0.73	3	6
**Body shape**	Long—Short	371	4.70	0.79	3	7
**Body shape**	Long—Short legged	372	4.80	0.76	3	7
**Body direction**	Uphill—Downhill	360	5.10	0.68	3	7
**Length of neck**	Long—Short	372	4.80	0.70	3	7
**Position of neck**	Vertical—Horizontal	370	4.70	0.81	3	7
**Shape of neck**	Arched—Straight	370	4.90	0.95	3	7
**Withers**	High—Low	372	4.90	0.73	3	7
**Position of shoulder**	Sloping—Straight	366	5.20	0.74	4	7
**Line of back**	Straight—Swayback	370	5.20	0.60	3	7
**Loins**	Long—Short	370	4.80	0.70	3	7
**Shape of croup**	Sloping—Straight	372	4.60	0.65	3	7
**Length of croup**	Long—Short	369	4.90	0.62	3	6
**Foreleg**	Over at knee—Back at knee	362	5.00	0.80	2	7
**Foreleg**	Toed in—Toed out	363	5.20	0.42	4	7
**Hind leg**	Sickle—Straight	351	5.00	0.52	3	6
**Hind leg**	Cow hocked—Bowlegged	370	5.00	0.65	3	7
**Correctness in movements**	Winging—Paddling	360	5.10	0.59	3	7
**Hooves**	Big—Small	360	5.00	0.42	3	7

**Table 4:** Assessment scale, number of observations, mean and standard deviation (SD) for the linear descriptive trait for jumping traits.

Variable	Scale	Horses	Mean	SD	Min	Max
**Take off—power**	Powerful—Weak	372	5.00	1.19	2	8
**Take off—quickness**	Quick—Slow	372	4.70	1.05	1	8
**Take off—direction**	Upwards—Forwards	372	4.80	1.16	2	8
**Technique: foreleg**	Bent—Hanging	372	4.60	1.05	2	8
**Technique: haunches**	Open—Tight	379	4.60	1.05	1	8
**Technique: back**	Rounded—Hollow	372	4.90	1.14	2	9
**Scope**	Upright—Weak	379	5.10	1.42	2	9
**Elasticity**	Elastic—Stiff	372	4.80	1.00	2	7
**Carefulness**	Too careful—Not careful	372	4.40	1.12	1	9
**Distance estimation**	Secure—Insecure	372	4.90	0.99	3	9

**Figure 2:**
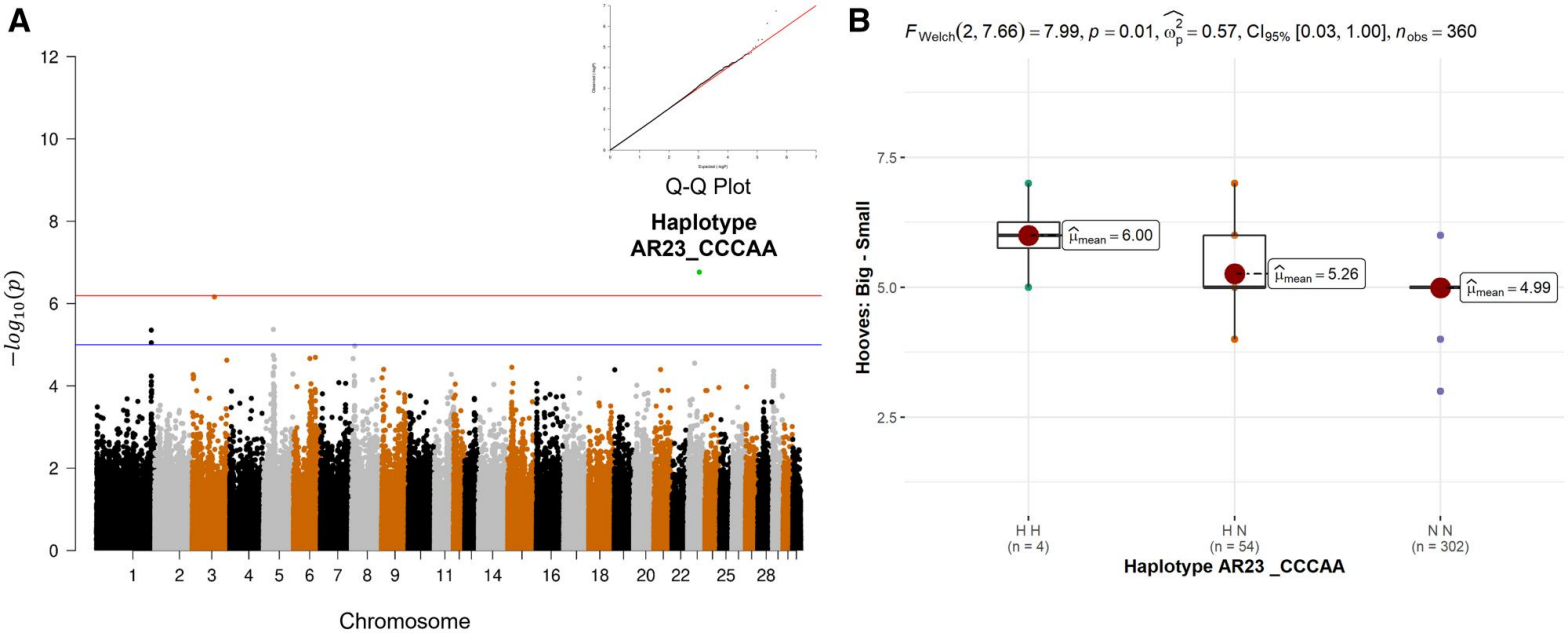
Genome-wide association study with haplotype blocks for the linear descriptive trait hooves. (A) Manhattan plot with the bottom line representing the suggestive significance threshold (*P* < 10^−6^) and the top line the significance threshold after Bonferroni correction (*P* < 6.4 × 10^−7^). The plot on the right-hand corner shows the quantile–quantile (Q–Q) plot with the observed plotted against the expected *P*-value. (B) Score for hooves for horses with the most significant haplotype (H) on chromosome 23, compared with for horses with other haplotypes (N) treated as bi-allelic variants. The red dot indicates the average value. Above the plot the results from the F-welch statistics are reported.

**Figure 3:**
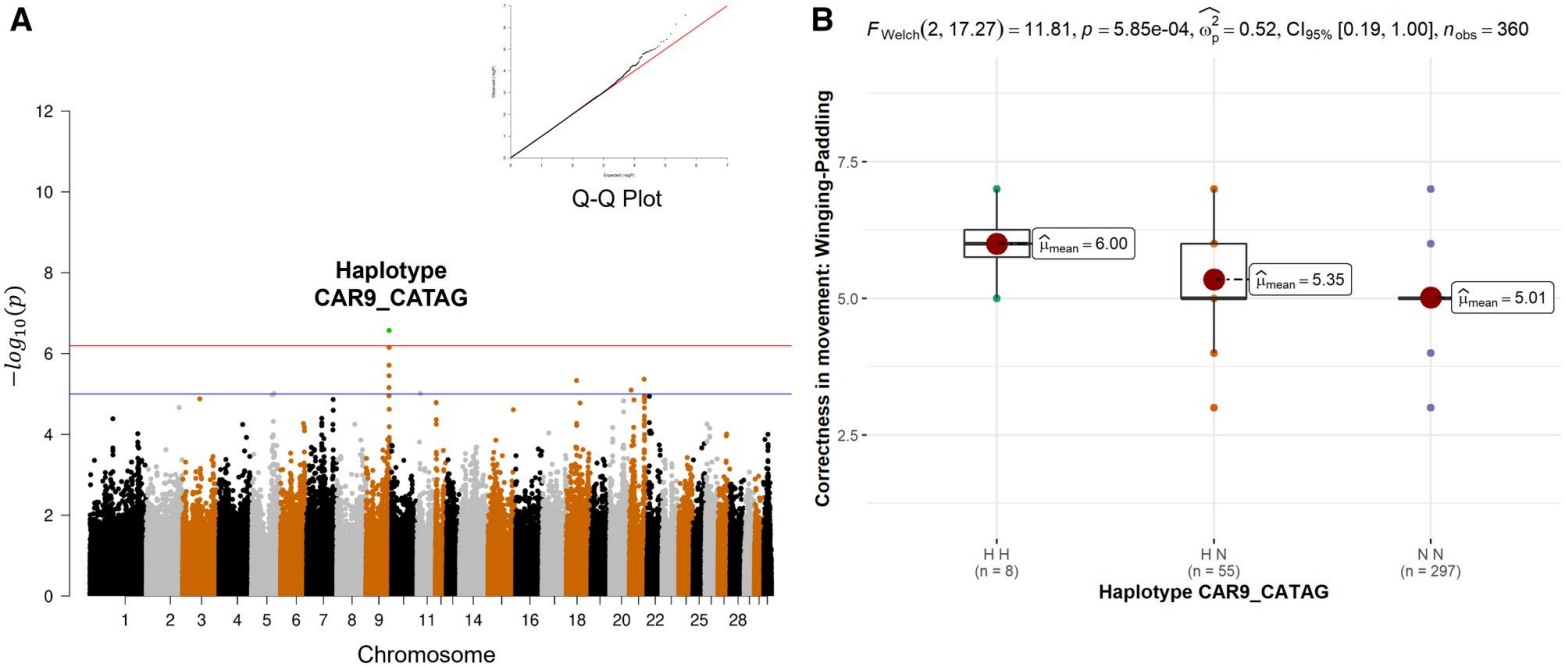
Genome-wide association study with haplotype blocks for the linear descriptive trait correctness in movements. (A) Manhattan plot with the bottom line representing the suggestive significance threshold (*P* < 10^−6^) and the top line the significance threshold after Bonferroni correction (*P* < 6.4 × 10^−7^). The plot on the right-hand corner shows the quantile–quantile (Q–Q) plot with the observed plotted against the expected *P*-values. (B) Score for correctness in movements of horses with the most significant haplotype (H) on chromosome 9, compared with other haplotypes (N), treated as bi-allelic variants. The red dot indicates the average value. Above the plot the results from the F-welch statistics are reported.

One haplotype on ECA23 (*P *= 1.84E-07) was found to be associated with the trait hooves (big–small). Horses homozygous for the AR23_CCCAA haplotype had smaller hooves (+1.01 scores) compared to horses not carrying this haplotype (*P*-value = 0.01) (Figure 2). A total of 18 genomic elements were present in the region of the AR23_CCCAA haplotype ± 500 kb: 10 protein coding genes, 7 IncRNA, and 1 scaRNA. One QTL in this region has previously been shown to be associated with gaits (Amano et al. 2018). A haplotype on ECA9 (*P *= 2.67E-07) was associated with correctness in movement (winging—paddling) (Table 5). Horses homozygous for the CAR9_CATAG haplotype had a significantly more paddling movement (+0.99 scores) compared to horses without this haplotype horses as shown in Figure 3 (*P*-value < 0.01). A total of nine genomic elements resided within ± 500 kb from the CAR9_CATAG haplotype: two protein-coding genes, four IncRNA, two miRNA, and one snRNA. Three QTLs found in this region have previously been reported to be associated with body weight, altitude adaptation, and temperament traits.

**Table 5:** Haplotype blocks significantly associated with five linearly described traits: two conformation and three free jumping traits **N_HH_** denotes the number of individuals carrying the HH haplotype. Annotated protein coding genes found within 500 kb from the haplotype are also shown.

Trait	Haplotype	ECA	Centered position	*P*-value	beta coefficient (SE)	N_HH_	Annotated protein coding genes
**Conformation**							
**Correctness in movements**	CAR9_CATAG	9	77521277	2.67E-07	0.39 (0.07)	8	*KHDRBS3, ZFAT*
**Hooves**	AR23_CCCAA	23	37901224	1.84E-07	0.32 (0.06)	4	*ACER2, ADAMTSL1, DENND4C, HAUS6, PLIN2, RPS6, RRAGA, SAXO1, SLC24A2*
**Free jumping**							
**Free jumping carefulness**	CAR24_AGACC	24	30271609	5.15E-08	0.80 (0.15)	3	–
	R26_TAATC	26	7442226	3.64E-07	0.69 (0.14)	6	–
**Free jumping distance estimation**	CAR10_AAATA	10	30904322	5.56E-07	0.45 (0.09)	32	*COL12A1, COX7A2, FILIP1, IMPG1, MYO6, SENP6, TMEM30A*
**Free jumping technique: haunches**	CAR10_AACTA	10	69572446	2.77E-07	−0.66 (0.13)	5	*TBC1D32*

**Figure 4:**
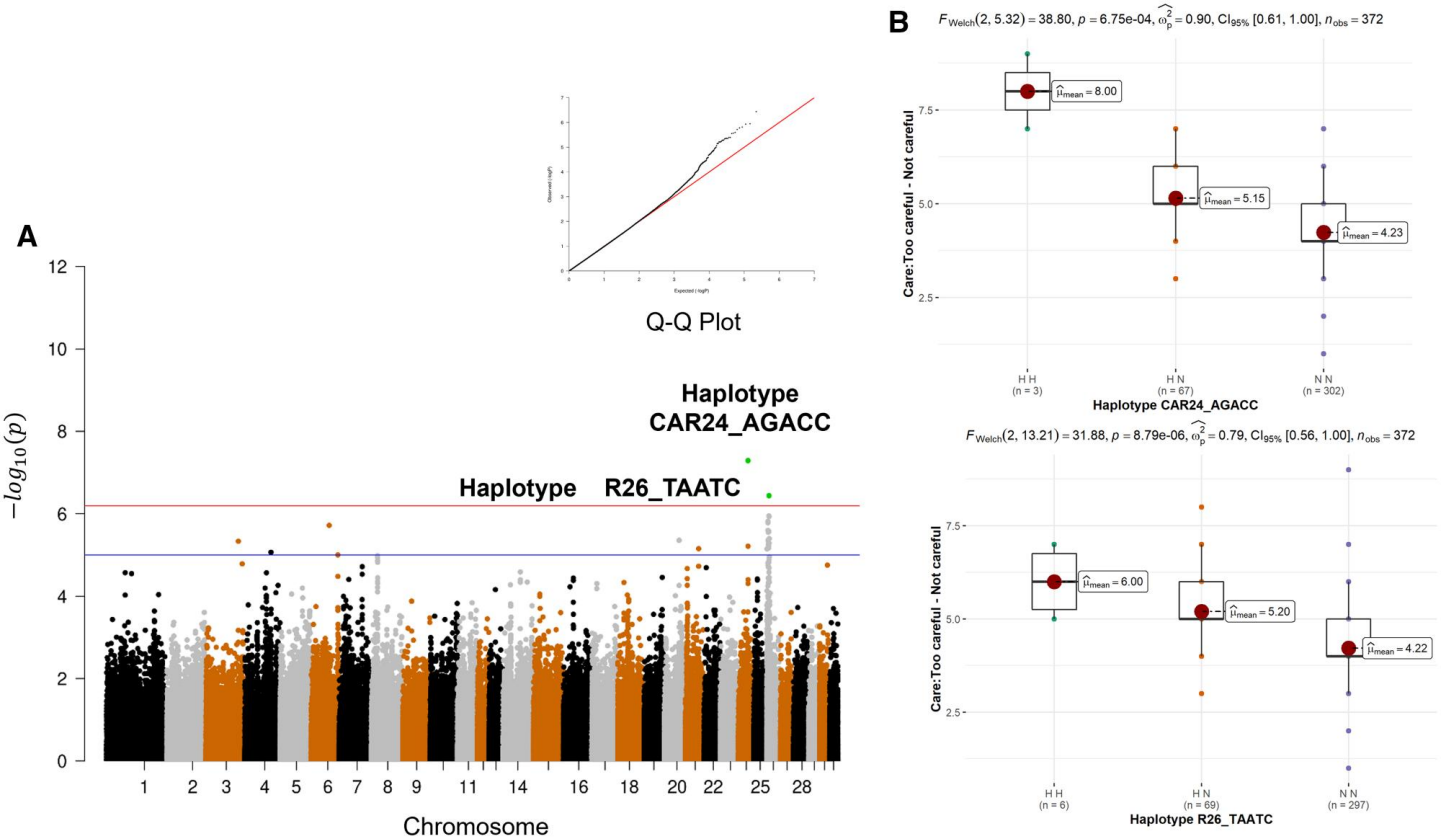
Genome-wide association study with haplotype blocks for carefulness. A) Manhattan plot (bottom line) representing the suggestive significance threshold (*P* < 10^−6^) and (top line) the significance threshold corrected for the effectively independent haplotype blocks, Bonferroni Correction (*P* < 6.4 × 10^−7^). The plot on the right-hand corner shows the quantile–quantile (Q–Q) plot with the observed plotted against the expected *P*-value. (B) Carefulness divided by the most significant haplotypes which have been treated as bi-allelic variants (H = haplotype allele and N = other N alleles). The red dot indicates the average value. Above the plot the results from the F-welch statistics are reported.

Two haplotypes, one on ECA24 (*P *= 5.15E-08) and one on ECA26 (*P *= 3.64E-07), were associated with free-jumping carefulness (too careful—not careful). Horses homozygous for either the CAR24 _AGACC or R26_TAATC haplotype were significantly less careful in free jumping compared with horses not carrying these haplotypes (*P*-value < 0.01) (+3.77 and +1.78 scores, respectively) (Figure 4). No protein coding genes were found in the vicinity of these haplotypes, but five IncRNA were found within the R26_TAATC haplotype (Supplementary Table S2). One QTL has previously been reported to be associated with insect bite hypersensitivity and overlapped the R26 _TAATC haplotype region (Schurink et al. 2012).

**Figure 5:**
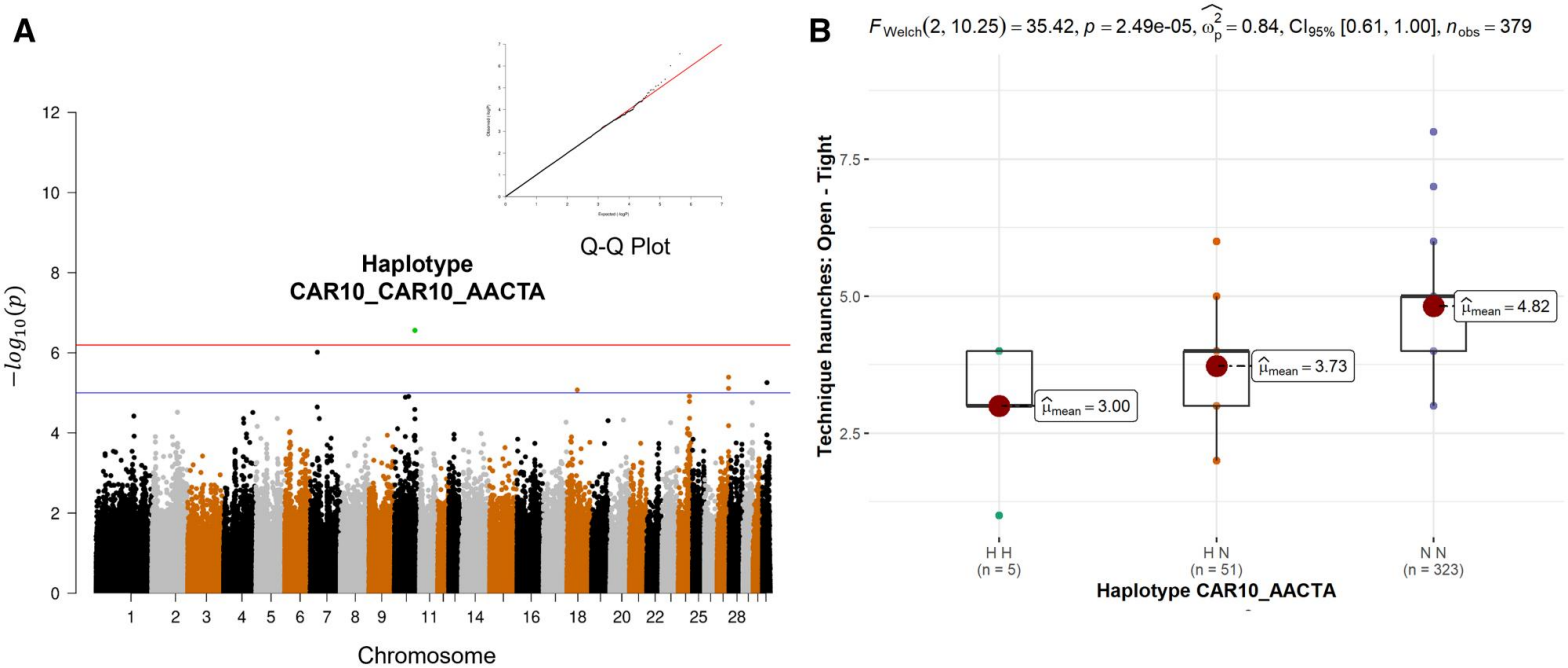
Genome-wide association study with haplotype blocks for technique haunches. (A) Manhattan plot (bottom line) representing the suggestive significance threshold (*P* < 10^−6^) and (top line) the significance threshold corrected for the effectively independent haplotype blocks, Bonferroni Correction (*P* < 6.4 × 10^−7^). The plot on the right-hand corner shows the quantile–quantile (Q–Q) plot with the observed plotted against the expected *P*-value. (B) Technique haunches divided by the most significant haplotype which has been treated as bi-allelic variants (H = haplotype allele and N = other N alleles). The red dot indicates the average value. Above the plot the results from the F-welch statistics are reported.

**Figure 6:**
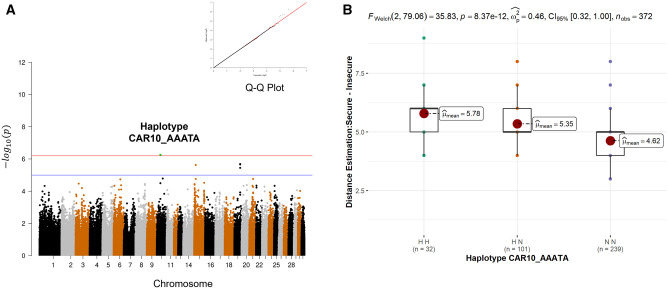
Genome-wide association study with haplotype blocks for distance estimation. (A) Manhattan plot (bottom line) representing the suggestive significance threshold (*P* < 10^−6^) and (top line) the significance threshold corrected for the effectively independent haplotype blocks, Bonferroni Correction (*P* < 6.4 × 10^−7^). The plot on the right-hand corner shows the quantile–quantile (Q–Q) plot with the observed plotted against the expected *P*-value. (B) Distance Estimation divided by the most significant haplotypes which has been treated as bi-allelic variants (H = haplotype allele and N = other N alleles). The red dot indicates the average value. Above the plot the results from the F-welch statistics are reported.

Also, two haplotypes on ECA10 (*P *= 2.77E-07 and *P *= 5.56E-07) were associated with free jumping technique: haunches (open–tight) and distance estimation (secure–insecure), respectively. Horses with the CAR10_AACTA haplotype had more open haunches (−1.82 scores) compared to NN horses (Figure 5). In the CAR10_AACTA haplotype ± 500 kb region one protein-coding gene, one pseudogene, and one IncRNA were found, as well as a QTL for height at withers. Horses with the CAR10_AAATA haplotype were significantly more insecure (+1.16 scores) compared to NN horses in the distance estimation (*P*-value < 0.01) (Figure 6). A total of 12 genomic elements were present from CAR10_AAATA haplotype ± 500 kb: eight protein-coding genes, four IncRNA, and one snRNA, whereas no QTLs overlapped this region.

## Discussion

A total of 380 horses were evaluated in this initial study; this number was sufficient to provide first insights, but limited the statistical power to detect associations, especially for traits influenced by multiple small-effect variants as performance-related traits. Nevertheless, based on the comparison between phenotypes in this subset and those presented in a larger cohort of SWB horses (>3,000) evaluated at the same age and with the same protocol, we found comparable means and variances (Viklund and Eriksson 2018). Therefore, we can affirm that the horses in this study were a fair representation of the phenotypic variability present at young horse test in the SWB breed. In addition, we employed a haplotype-based GWAS approach, which could be a noteworthy advancement over traditional single SNP-based methods since it helps in the detection of aggregated genetic effects. By analyzing haplotypes, and therefore the combination of alleles at adjacent loci, it is possible to capture more genetic variation and potentially identify more relevant associations (Barendse 2011; Bovo et al. 2021). Although this method has not been widely applied in literature yet, there are some interesting insights into the potential of this method. Barendse (2011) highlighted that haplotype analysis provided stronger evidence for candidate genes related to intramuscular fat percentage in cattle and might be a useful tool to reduce the missing heritability issue. Indeed, haplotypes reflect the inheritance of contiguous genomic regions, making them biologically more relevant than individual SNPs (Lorenz et al. 2010).

Up to date, in horses a SNP-based GWAS approach has been mostly used for identifying genetic variants associated with specific traits. One of the most prominent examples was the identification of the effect of *MSTN* gene polymorphisms on performance in Thoroughbreds. Hill et al. (2010) discovered a SNP (g.66493737C>T) in the *MSTN* gene strongly associated with racing distance in thoroughbreds. Furthermore, the same authors extended these findings by showing that this SNP also has predictive potential for speed (Hill et al. 2012). This example highlights the potential of GWAS to reveal meaningful associations; however, it also underscores the rarity of large-effect variants in equine performance. Most traits relevant to sport horses, such as jumping ability, correctness of movements, and conformation, are most likely complex and polygenic, probably governed by the cumulative action of many variants with small effects. In such cases, traditional SNP-based GWAS approaches often do not provide expected results, particularly in studies with limited sample sizes or modest phenotypic variability. Furthermore, single-SNP analyses do not account for the linkage disequilibrium (LD) structure of the genome and may miss biologically meaningful signals that are only detectable when considering the combined effects of multiple nearby variants (Lorenz et al. 2010). This limitation provides the rationale for exploring alternative approaches such as haplotype-based GWAS, which can offer a more powerful and biologically informative framework for detecting associations in complex traits. Thus, we decided to employ a haplotype-based GWAS approach to study performance traits in SWB horses. In addition, since a previous study found a larger genetic variation in jumping traits compared to movements among the tested horses at young horse evaluation (Viklund and Eriksson 2018), we decided to focus on conformation and jumping traits and exclude movement traits that would likely require a larger sample size. From a methodological point of view, we acknowledge that the block-based Bonferroni correction, which was chosen due to the strong correlation among haplotypes within blocks, did not fully account for the number of haplotypes tested within each block. Consequently, the detected associations should be interpreted as exploratory and require validation in larger, independent datasets. Nevertheless, the achieved Bonferroni-corrected significance threshold of *P* < 6.4 × 10^−7^ is in line with previous literature in horses employing medium- to high-density genotyping arrays (Kulbrock et al. 2013; Velie et al. 2018; Norton et al. 2019). Finally, although a MAF threshold of 0.01 was used to retain potentially informative haplotypes, rare haplotypes with few homozygous carriers need further validation. The relatively large number of haplotype blocks used in this study (78,000) reflects the underlying LD structure in this population. The horses in this study originate from the semi-open SWB studbook, which imports genetic material from all over Europe, resulting in more rapid LD decay compared to highly inbred or closed populations. A very low average genomic inbreeding coefficient was previously estimated within SWB horses (Ablondi et al. 2019b). In such an outbred population, long haplotype blocks tend to fragment into many rare haplotypes, leading to sparse genotype classes and reduced statistical power. Therefore, short haplotype windows of 5 SNPs were chosen as a compromise to capture local multi-marker LD while maintaining sufficient haplotype frequencies for association testing. Given the post-QC marker density (roughly 390K SNPs across the autosomes), a 5-SNP window corresponds on average to approximately 25 kb, which aligns well with the scale of local LD observed in this population. As a result, the potential advantage of haplotype-based GWAS in this study might not be related to capturing long-range haplotype blocks, but rather to aggregating the effects of multiple correlated variants within local LD regions.

We identified several significant haplotypes associated with height at withers and free-jumping traits. For height at withers, a total of 33 genomic elements were found within ± 500 kb from the five significantly associated haplotypes. These elements likely play regulatory roles, influencing gene expression and phenotype appearance. Specifically, the *LCORL* gene, located within one of those significant regions, has been implicated in growth and body size regulation in various species, including horses (Makvandi-Nejad et al. 2012; Rubin et al. 2012; Tetens et al. 2013). The haplotype with the most pronounced effect for height at withers was the CAR3_B3033_TGACA haplotype, resulting in an average increase of 3.78 centimeters. This finding might be particularly relevant for breeders aiming to breed horses with specific height requirements. For correctness of movement, a gene of interest was the *KHDRBS3*, which encodes an RNA-binding protein that regulates alternative splicing of genes involved in synaptic function, including *NRXN1*, which is critical for excitatory synapse formation and plasticity. Given its expression in the hippocampus and other neuronal tissues, *KHDRBS3* may contribute to the fine motor skills and adaptive responses required during the correctness of movements.

Regions associated with free jumping traits, such as the CAR10_AAATA haplotype, significant for distance estimation, and the CAR10_AACTA haplotype, significant for technique in haunches, highlighted potential interesting candidate genes. Most of these genes located in those regions share biological functions in musculoskeletal structure, energy metabolism, and neuromuscular coordination—elements that might have an important role in successfully jumping. The *COL12A1*, encoding a fibril-associated collagen, contributes to the organization of the extracellular matrix in muscle and tendon tissues. Mutations in *COL12A1* have been linked to a spectrum of myopathic conditions in humans, including Ehlers-Danlos syndrome, where phenotypes such as joint hypermobility, delayed musculoskeletal development, and muscle hypotonia are observed (Zou et al. 2014). Such findings support the hypothesis that *COL12A1* variation may affect the mechanical properties and elasticity of the locomotor system in horses, ultimately influencing jumping ability. In addition, *MYO6* deserves particular attention due to its role in muscle contraction. This gene contributes to the maintenance of cellular architecture and vesicle trafficking—processes that are critical during repeated muscle contractions and cellular stress induced by athletic performance. The gene *MYO6* has also been implicated in maintaining the stability of neuromuscular junctions (Osterweil et al. 2005), suggesting that it may influence signal transmission between nerves and muscles and thereby contribute to coordination and power in free jumping tasks. Although its specific role in equine muscle or performance has yet to be characterized, its conserved function across species highlights it as a biologically plausible contributor to the complex neuromuscular demands of jumping.

Moreover, the gene *FILIP1*, also in a region associated with free jumping, is implicated in actin cytoskeleton organization and has been shown to regulate myogenic differentiation, particularly in regenerating muscle fibers. A recent study proposed that *FILIP1*-mediated homeostasis of filamins is required for the proper development of skeletal and cardiac muscle cells and likely also their maintenance under conditions of sustained mechanical stress (Reimann et al. 2020). The presence of genes related to connective tissue structure and muscle functions further corroborates results from previous studies based on the genomic signature of selection in the SWB horses, where the importance of high mobility and relaxed locomotion patterns was highlighted (Ablondi et al. 2019a, 2019b). This haplotype-based GWAS study provides novel insights into the genetic architecture of height at withers and free jumping traits in SWB horses. Despite the limitation of a modest sample size, our findings highlight the potential of haplotype analysis in uncovering relevant genetic associations with a practical implementation in breeding programs. Future studies with larger sample sizes, coupled with novel sources for objective phenotyping enabled by emerging Artificial Intelligence approaches to image and video analyses, will be essential for elucidating the genetic mechanisms underlying sporting performance. Such advances may in the future allow more accurate and comprehensive characterization of equine performance traits, in addition to broadening the knowledge of the genetic mechanisms behind sport performance.

## Conclusion

This study represents the first application of a haplotype-based GWAS approach to evaluate complex performance traits in SWB horses, revealing several biologically meaningful associations despite a modest sample size. Notably, beyond the expected association between the *LCORL* locus and height at withers, we identified haplotypes associated with free jumping traits that encompass genes of functional relevance. The *COL12A1*, involved in extracellular matrix integrity, and *FILIP1*, which regulates muscle differentiation and response to mechanical load, suggest a genetic basis underpinning muscle elasticity and strength: key components of jumping performance. Furthermore, the identification of *MYO6*, a gene implicated in intracellular trafficking and neuromuscular junction stability, provides a novel insight into the molecular mechanisms that may affect coordination. These findings not only support the biological plausibility of the detected associations but also pinpoint promising candidate genes for further functional validation. By highlighting these specific loci, this study offers a concrete contribution to the understanding of the genetic architecture of equine athleticism, with particular emphasis on free jumping performance. In future studies, increasing the sample size and integrating more objective phenotyping, potentially through video-based AI analyses, will be crucial to validate these associations and explore their predictive value in breeding programs.

## Supplementary Material

skag109_Supplementary_Data

## Data Availability

The datasets in the current study were generated and analyzed in collaboration with the Swedish Warmblood Association, and have a commercial value for them. Thus, the SWB horse data are available from the corresponding author on reasonable request.

## Abbreviations

*ACOD1*: aconitate decarboxylase 1
*CCL5*: C-C motif chemokine ligand 5
*CD40LG*: CD40 ligand
*COL12A1*: collagen type XII alpha 1 chain
*CNTN6*: contactin 6
*DCC*: deleted in colorectal carcinoma
*DEG*: differentially expressed gene
*DMRT3*: doublesex and mab-3 related transcription factor 3
*FILIP1*: filamin A interacting protein 1
*FOS*: Fos proto-oncogene, AP-1 transcription factor subunit
GHap: Genome-wide Haplotype (R package)
GS: genomic selection
GWAS: genome-wide association study
HWE: Hardy–Weinberg equilibrium
*IL1R2*: interleukin 1 receptor type 2
*IL20RA*: interleukin 20 receptor subunit alpha
*IL22RA2*: interleukin 22 receptor subunit alpha 2
*IncRNA*: long intergenic non-coding RNA
*KCNIP4*: Kv channel interacting protein 4
*KHDRBS3*: KH RNA binding domain containing, signal transduction associated 3
*LCORL*: ligand dependent nuclear receptor corepressor like
MAS: marker-assisted selection
MAF: minor allele frequency
miRNA: microRNA
*MSTN*: myostatin
*MYO6*: myosin VI
*NIPA1*: non-imprinted in Prader-Willi/Angelman syndrome 1
*NRXN1*: neurexin 1
QC: quality control
Q-Q: quantile–quantile
QTL: quantitative trait locus/loci
*RELN*: reelin
*SHANK2*: SH3 and multiple ankyrin repeat domains 2
SHAPEIT: Segmented HAPlotype Estimation and Imputation Tool
SNP: single nucleotide polymorphism
snRNA: small nuclear RNA
*STAU2*: staufen double-stranded RNA binding protein 2
SWB: Swedish Warmblood Breed
